# Mitochondrial metabolic reprogramming by SIRT3 regulation ameliorates drug resistance in renal cell carcinoma

**DOI:** 10.1371/journal.pone.0269432

**Published:** 2022-06-07

**Authors:** Young-Ran Gu, Jinu Kim, Joon Chae Na, Woong Kyu Han

**Affiliations:** 1 Department of Urology, Urological Science Institute, Yonsei University College of Medicine, Seoul, Korea; 2 Center of Uro-Oncology, Yonsei Cancer Hospital, Seoul, Korea; Facultad de Estudios Superiores Iztacala, Universidad Nacional Autónoma de México, MEXICO

## Abstract

Clear cell renal cell carcinoma (ccRCC) alters metabolic signals frequently, leading to mitochondrial dysfunction, such as increase of glycolysis and accumulation of lipid. Sirtuin3 (SIRT3) is a key factor for the regulation of both mitochondrial integrity and function. SIRT3 is downregulated and contributes in both cancer development and progression in ccRCC. The aim of this study is to investigate SIRT3-regulated mitochondrial biogenesis in ccRCC. SIRT3 overexpression alone reduced glucose uptake rate and enhanced membrane potential in mitochondria. ccRCC with overexpressed SIRT3 further improved the lethal effects when combined with anticancer drugs (Resveratrol, Everolimus and Temsirolimus). Cell viability was markedly decreased in a dose-dependent manner when treated with resveratrol or mTOR inhibitors in SIRT3 overexpressing ccRCC. In conclusion, SIRT3 improved mitochondrial functions in ccRCC through metabolic reprogramming. Mitochondrial reprogramming by SIRT3 regulation improves the sensitivity to anticancer drugs. The combination of SIRT3 and resveratrol functioned synergistically lethal effect in ccRCC.

## Introduction

Clear cell renal cell carcinoma (ccRCC) is the most common and aggressive type of kidney cancer. Signaling networks that regulate metabolic behavior in ccRCC are frequently altered, leading to ’metabolic disease’ tumors [1] that characteristically exhibit a Warburg phenotype (a cancer hallmark), mitochondrial dysfunction, and elevated fat deposition [2]. Metabolic reprogramming toward low mitochondrial oxidative phosphorylation (OXPHOS) is also a prominent feature of ccRCC, resulting from decreased mitochondrial DNA, respiratory-chain activity, and oxidative phosphorylation-related proteins [3]. In approximately 90% of cases, this metabolic reprogramming in ccRCC is often related to von Hippel-Lindau (VHL) tumor-suppressor mutations; in VHL-deficient ccRCC, HIF1α increases the expression of GLUT-1, which then promotes cellular glucose uptake [4]. Despite the acknowledged importance of metabolic alterations in ccRCC tumorigenesis, the molecular mechanisms underlying these metabolic shifts are incompletely understood.

Mitochondria are key organelles that generate ATP through oxidative phosphorylation. However, they perform other roles beyond energy production, including reactive oxygen species (ROS) generation, cell signaling, cell-death regulation, and biosynthetic metabolism. Mitochondria also influence cancer initiation, growth, survival, and metastasis under harsh conditions, such as during nutrient depletion, hypoxia, and during cancer treatments [5]. The Warburg effect, a type of metabolic switch favoring aerobic glycolysis/lactic acid fermentation, is observed in most cancer cells and is especially enhanced in high-grade ccRCC tissues [6]. These ccRCC tissues also have reduced mitochondrial content, as well as defective mitochondrial structures and activities [7].

Sirtuins (SIRT) are a conserved family of NAD-dependent ADP-ribosyltransferases and/or protein deacetylases. The family consists of seven members (SIRT1-7) in mammals, and each is involved in metabolism, stress responses, and longevity [8]. Among these, SIRT3 is localized to mitochondria and is a crucial factor for the regulation of both mitochondrial integrity and function. SIRT3 activates enzymes involved in mitochondrial fuel catabolism [9] and mediates peroxisome proliferator-activated receptor gamma coactivator-1 alpha (PGC1α), affecting both cellular ROS production and mitochondrial biogenesis [10]. In addition, SIRT3 physically interacts with and regulates complexes I, II, and V of the electron transport chain of the inner mitochondrial membrane [11]. Interestingly, SIRT3 can also function as a pro-apoptotic signal in some cancer cells, acting as a tumor-suppressing cell guard.

In ccRCC tissues, SIRT3 is downregulated [2] and regulates the Warburg effect by shifting cancer-cell metabolism by destabilizing HIF1α [12]. Moreover, reduced SIRT3 activity contributes to oxidative stress in mitochondria [13]. Therefore, the contribution of SIRT3 to ccRCC is as a tumor suppressor in both its development and progression. The transcriptional coactivator PGC1α is a key regulator of mitochondrial biogenesis through its interactions with numerous transcription factors [14]. PGC1α is highly expressed in a variety of biological pathways, in mitochondria, and in other tissues involved in energy metabolism and mitochondrial biogenesis. Recently, PGC1α has been linked to cancer progression, proliferation, invasiveness, and metastasis, with PGC1α-dependent mitochondrial biogenesis contributing to tumor metastatic potential [5]. PGC1α promotes fatty-acid oxidation and glucose-derived lipogenesis in cancer [14].

Here, we investigated SIRT3-regulated mitochondrial biogenesis in ccRCC. We focused on: (1) genes related to mitochondrial function that are affected by SIRT3 overexpression, (2) SIRT3-related normalization of mitochondrial function, and (3) the potential for the development of SIRT3 as a therapeutic agent. The results suggest the possibility that SIRT3 may function as a novel therapeutic agent for human ccRCC by regulating mitochondrial function.

## Materials and methods

### Cell culture and chemicals

The human kidney carcinoma cell lines Caki-1 and 786-O and normal human kidney tubule cell HK2 and Primary Renal Proximal Tubule Epithelial Cell (RPTEC) were obtained from American Type Culture Collection (ATCC, Manassas, VA, USA). The Caki-1 cell was cultured in McCoy’s 5A medium (Gibco, Grand Island, NY, US) and the 786-O cell was maintained in RPMI 1640 medium (Hyclone, Logan, UT, US). McCoy’s 5A and RPMI 1640 media were supplemented with 10% fetal bovine serum (FBS) and 1% penicillin/streptomycin at 37°C with 5% CO2. HK2 cell was cultured in Keratinocyte Serum Free Medium (K-SFM) supplemented with Keratinocyte-SFM Supplements (Gibco, Grand Island, NY, US). RPTEC was cultured in Renal Epithelial Cell Basal Media supplemented with renal epithelial cell growth kit components (ATCC Manassas, VA, USA). The identity of every cells was confirmed using STR profiling from ATCC. For the SIRT3 overexpression experiments, cells were transfected with the pcDNA3-SIRT3-FLAG vector (Addgene, Watertown, MA, US) or the pcDNA3-FLAG empty vector using Lipofectamine 2000 transfection reagent (Invitrogen, Carlsbad, CA, US). After transfection (48 hr) with the SIRT3 expression vector, medium was supplemented with either DMSO or drugs for an additional 48 hr. Trans-resveratrol (5-[(1E)-2-(4-hydroxyphenyl)ethenyl]-1,3-benzenediol) was purchased from Cayman Chemical (Ann Arbor, MI, US), and everolimus and temsilorimus (CCI-779) were purchased from Selleckchem (Houston, TX, US).

### Quantitative real-time PCR (qRT-PCR)

Total RNA was extracted using TRI Reagent (Invitrogen) and then reverse transcribed using EasyScript Reverse Transcriptase (Transgen Biotech, Beijing, CN). The resulting cDNA was amplified and qRT-PCR was performed using SYBR TOPreal qPCR 2× PreMIX (Enzynomics, Daejeon, KR) using the StepOnePlus system (Applied Biosystems, Foster city, CA, US). All procedures were performed according to the manufacturer’s instructions. cDNA sample analyses were performed in triplicate.

### Western blotting

Cells were washed twice using ice-cold PBS and then lysed on ice using 1× RIPA lysis buffer (Thermo Fisher Scientific, Waltham, MA, US) containing freshly added 1× protease and phosphatase inhibitors (Thermo Fisher Scientific). After incubation on ice (30 min), cell lysates were collected by centrifugation at ~14,000 x g for 15 min at 4°C. Protein concentrations were determined using a bicinchoninic acid assay (BCA) protein assay kit (Pierce, Rockford, IL, US). Equal amounts of total protein were loaded onto sodium dodecyl sulfate-polyacrylamide gels for separation and then transferred to polyvinylidene fluoride membranes. Membranes were incubated with PGC1α (Abcam, Cambridge, UK), SIRT3 (Cell Signaling Technology, Danvers, MA, US), PHD3 (Novus, Centennial, CO, US) and β-actin (Santa Cruz Biotechnology, Dallas, TX, US) primary antibodies diluted 1:1,000 with 5% BSA Tris-buffered saline-Tween 20 (TBS-T). HRP-conjugated secondary antibodies and West Dura chemiluminescence kits (Thermo Fisher Scientific) were used for protein-band detection.

### Cell viability assay (CCK-8 assay)

786-O and Caki-1 cells in 96-well culture plates were treated with different doses of anticancer drugs for 48 hr. Cell viability was determined using a Cell Counting Kit-8 (CCK-8, Enzo Life Sciences, Farmingdale, NY, US). All procedures were performed according to the manufacturer’s instructions. Cells were incubated for 2 hr at 37°C, and viability was determined using a Beckman Coulter microplate signal reader at 450 nm.

### Mitochondrial membrane-potential assay

Cells in black 96-well culture plates were treated with the mitochondrial probe 5,5’,6,6’-tetrachloro-1,1’,3,3’-tetraethyl benzimidazolyl-carbocyanine iodide (JC-1, Cayman Chemical) and incubated at 37°C for 30 min. Cells were analyzed using a Varioskan Flash 3001 fluorescence plate reader (Thermo Fisher Scientific) and all procedures were performed according to the manufacturer’s instructions.

### Confocal microscopy

Caki-1 and 786-O cells were grown and transfected as described above using 4-well culture slide chambers (BD Falcon, Bedford, MA, US). After transfection (48 hr) with the SIRT3 expression or empty vector, culture media was removed and replaced with media containing 100 nM MitoTracker CMXRos (Invitrogen) added to the live cells for 15 min at 37°C. Images of the cells were taken using a confocal microscope (LSM 700, Carl Zeiss, Oberkochen, Germany) and analyzed using LSM Image Browser software.

### Glucose uptake ratio assay

Glucose uptake (2-NBDG, a fluorescently tagged deoxyglucose analog) in Caki-1 and 786-O cells was measured using a glucose uptake cell-based assay kit (Cayman Chemical). In brief, cells in black 96-well culture plates were treated with drugs in glucose-free media for 48 hr, and then 2-NBDG was added for an additional 2 hr. The final concentration of 2-NBDG in the culture media is 100 ug/ml. Glucose uptake was measured by the uptake of 2-NBDG in glucose-free media, using a fluorescence plate reader (Thermo Fisher Scientific) at according to the manufacturer’s instructions.

### Glycolysis assay

786-O and Caki-1 cells were transfected with SIRT3 expression or empty vector and transferred in 96-well culture plates. After treated with drugs for 48 hr, glycolytic activity was determined by measuring the total amount of L-lactate, without correcting for lactate derived from glutaminolysis and glycogen degradation, the end product of glycolysis. L-Lactate standard and supernatant from each sample were measured using a Beckman Coulter microplate signal reader at 490 nm according to the manufacturer’s instructions (Cayman Chemical).

### Complex I enzyme activity assay

786-O and Caki-1 cells were transfected with SIRT3 and treated with drugs for 48 hr. Sample protein concentrations from 786-O and Caki-1 were determined using a bicinchoninic acid assay (BCA) protein assay kit (Pierce, Rockford, IL, US). Mitochondrial OXPHOS Complex I enzyme activity was measured using a Beckman Coulter microplate signal reader at 450 nm according to the manufacturer’s instructions (Abcam).

### Statistical analysis

All images are representative of at least three independent trials. Quantitative values are expressed as mean ± SEM. Statistical differences between groups were compared using Student’s t-tests and Mann-Whitney U tests using SPSS software version 23.0 (IBM SPSS Statistics, IBM Corp., Armonk, NY, US). A p value < 0.05 was considered statistically significant.

## Results

### SIRT3 overexpression upregulated genes related to mitochondrial biogenesis

**Fig 1:**
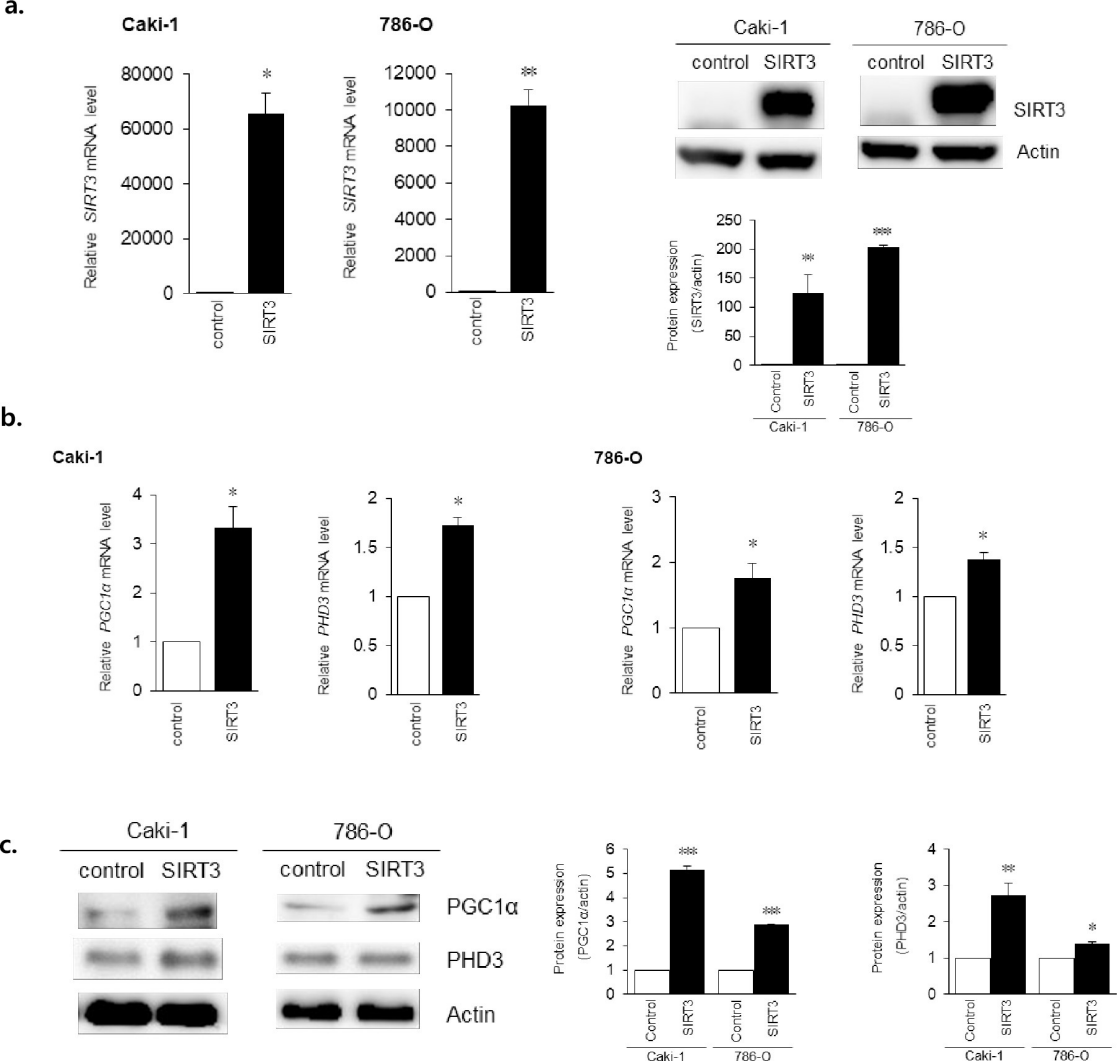
SIRT3 overexpression increased mitochondrial biogenesis in ccRCC cells. a. SIRT3 mRNA and protein levels were assessed using qRT-PCR and western blotting after SIRT3 overexpression (C: empty vector, S: SIRT3 expression vector). Values are expressed as mean ± SEM (n = 3, per triplicate) and control-cell values were set to 1. b. qRT-PCR was used to assess the mRNA levels of PGC1α and PHD3 after SIRT3 overexpression. Values are expressed as mean ± SEM (n = 5, per triplicate) and control-cell values were set to 1. *p <0.05, **p <0.01. c. Western blotting was used to assess PGC1α and PHD3 protein levels after SIRT3 overexpression. Representative images from three replicates. Quantification of proteins ratio obtained from triplicate samples and normalized onto β-Actin and represented in the bars graph. *p <0.05, **p <0.01, ***p <0.001.

To address whether SIRT3 overexpression affects mitochondrial biogenesis, we examined the expression levels of genes related to it. ccRCC and normal tubule cells were transfected with either a SIRT3-overexpression vector or an empty vector. Total RNA and protein were extracted from the transfected ccRCC cells, and SIRT3 mRNA and protein were assessed using qRT-PCR and western blotting (Fig 1A). mRNA levels for PGC1α, a master regulator of mitochondrial biogenesis, and PHD3, a regulator of glucose metabolism, were significantly increased by SIRT3 overexpression in 786-O and Caki-1 cells (Fig 1B). The 786-O cell is defective in VHL expression, significantly increased VHL mRNA level was measured with SIRT3 overexpression. While Caki-1 has wild-type *vhl*, mRNA levels of VHL did not change after SIRT3 overexpression (S1A Fig). In addition, PGC1α and PHD3 protein levels were also increased in SIRT3-overexpressing (OE) cells compared to controls (Fig 1C).

### Mitochondrial function improved after SIRT3 overexpression

**Fig 2:**
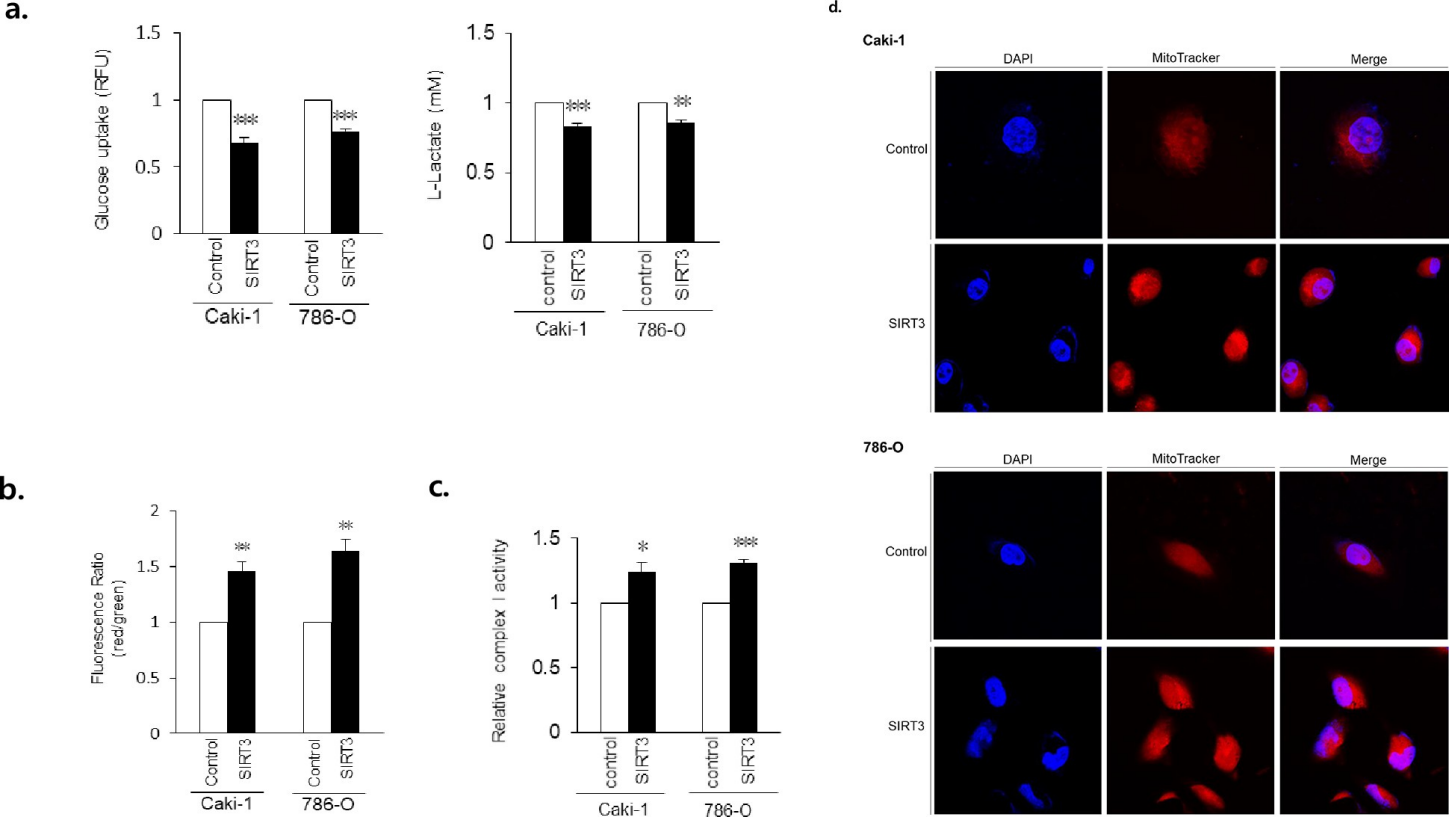
SIRT3 overexpression regulates mitochondrial function. a. Quantitative analysis of cellular glucose uptake (n = 6, per triplicate) and L-lactate production (n = 3, per triplicate). Values are expressed as mean ± SEMand control-cell values were set to 1. b. Mitochondrial membrane potentials were improved by SIRT3 overexpression. Data are expressed as mean ± SEM (n = 4, per triplicate) and control-cell values were set to 1. **p <0.01, ***p <0.001. c. Quantitative analysis of complex I enzyme activity assay. Values are expressed as mean ± SEM (n = 3, per triplicate) and control-cell values were set to 1. *p <0.05. d. Representative confocal ccRCC images after SIRT3 overexpression (objective magnification 63×). Mitochondria were stained using MitoTracker Red CMSRos for 15 min prior to fixation and then subjected to DAPI staining.

To determine whether SIRT3 overexpression (OE) improves mitochondrial biogenesis, we assessed glucose uptake and mitochondrial membrane potentials. As expected, SIRT3 OE cells showed a decrease in glucose uptake and enhanced mitochondrial membrane potentials. As L-lactate production is decreased, glycolysis is also reduced in SIRT3 OE RCC cells (Fig 2A and 2B). However normal tubule cells have no significant change with mitochondrial function, even though after SIRT3 overexpression (S2 Fig). Besides, we performed complex I enzyme activity assay to determine mitochondrial oxidative phosphorylation (OXPHOS). SIRT3 OE ccRCC cells showed an increase in complex I enzyme activity (Fig 2C). In addition, SIRT3 OE resulted in significantly elevated mitochondrial mass compared to controls (Fig 2D). Taken together, these data suggest that SIRT3 OE restores dysfunctional mitochondria, resulting in higher levels of healthy mitochondria.

### SIRT3 OE increased the cytotoxic effect of anti-cancer drug in cancer cell

**Fig 3:**
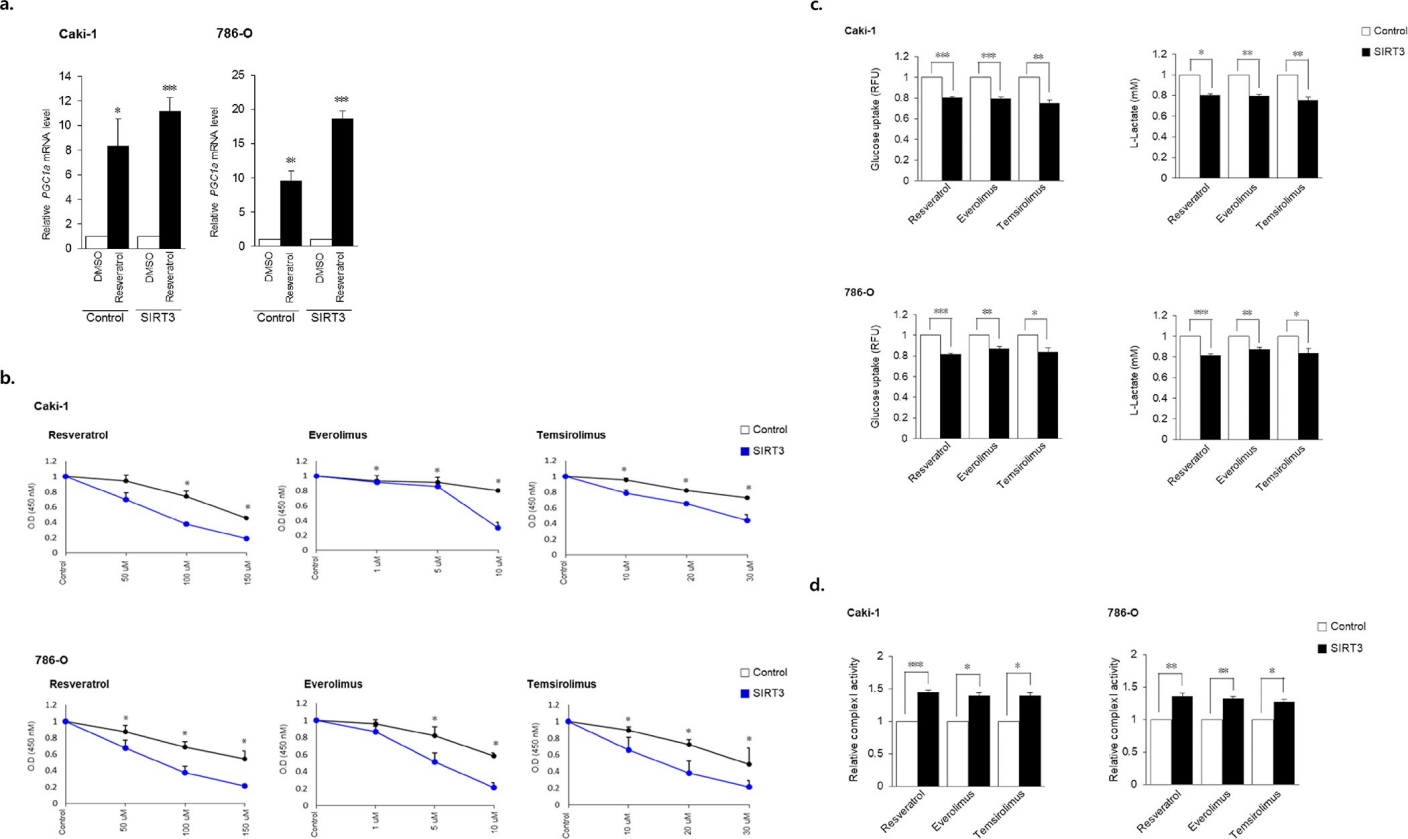
SIRT3 overexpression and resveratrol treatment improved PGC1α expression in ccRCC cells. a. SIRT3-overexpressing cells were treated with RSV. PGC1α mRNA levels were quantified using qRT-PCR. Values are expressed as mean ± SEM (n = 4, per triplicate) and control-cell values were set to 1. b. Anticancer drugs induce anti-proliferative effects and mitochondrial function in ccRCC cells (Black line: empty vector, blue line: SIRT3 expression vector). The proliferation of ccRCC cells was determined using a CCK-8 assay kit. Cells were treated with drugs for 48 hr after SIRT3 overexpression. Data are expressed as mean ± SEM (n = 5, per triplicate) and compared to controls. c. Glucose uptake (n = 4, per triplicate) and L-lactate production (n = 3, per triplicate)in ccRCC cells with and without drug treatments. Cells were treated with drugs for 48 hr after SIRT3 overexpression. Data are expressed as mean ± SEM and control-cell values were set to 1. *p <0.05, **p <0.01, ***p <0.001. d. Quantitative analysis of mitochondrial complex I enzyme activity in ccRCC cells with and without drug treatments. Data are expressed as mean ± SEM (n = 3, per triplicate) and control-cell values were set to 1. *p <0.05.

As resveratrol (RSV) improves mitochondrial biogenesis [15, 16], we assessed the relationship between SIRT3-improved mitochondrial function and RSV in RCC cells. We detected significantly elevated PGC1α mRNA levels in RSV-treated SIRT3 OE cells (Caki-1 p = 0.00000, 786-O p = 0.00004) compared to untreated SIRT3 OE cells (Fig 3A). Thus, SIRT3 and RSV functioned synergistically in ccRCC. When we investigated the cytotoxic effect of anti-cancer drug (mTOR inhibitor, RSV) in SIRT3 OE cancer cell, it showed the number of cancer cell was significantly decreased in a dose-dependent manner (Fig 3B). Additionally, both glycolytic activity and mitochondrial complex I enzyme activity were confirmed in SIRT3 OE RCC cells. RSV-treated SIRT OE ccRCC cells showed reduced glucose uptake and L-lactate production compared to untreated SIRT3 OE cells (Fig 3C). Furthermore, enhanced mitochondrial complex I enzyme activity was observed in drugs-treated SIRT3 OE ccRCC cells compared to untreated SIRT3 OE cells (Fig 3D). Thus, overexpression of SIRT3 enhanced the sensitivity of these cells to anticancer drugs.

## Discussion

Here, we observed marked suppression of SIRT3 expression in RCC cells and that SIRT3 restoration led to the recovery of impaired mitochondrial functions. These types of abnormal mitochondrial functions can cause many diseases, including cancer. In addition, such metabolic dysregulations caused by oncogene and tumor suppressor gene mutations are hallmarks of cancer and may provide opportunities for the development of cancer diagnostics, prognostics, and therapeutics [17, 18]. The six cancer concepts important to the present work are: (1) self-sufficient growth signaling, (2) evasion of growth suppressors, (3) cell-death resistance, (4) replicative immortalization, (5) angiogenesis, and (6) invasion and metastasis [19].

Among various cancers, ccRCC cell metabolism has been well-studied and has revealed many reprogrammed metabolic pathways. The primary molecular alteration reported in ccRCC is VHL inactivation (located on chromosome 3p) leading to the constitutive activation of HIFs through the stabilization of HIF subunits [1, 4, 20]. Caki-1 and 786-O cell are widespread cell line of ccRCC. While Caki-1 has wild-type *vhl*, it was shown to produce ccRCC in nude mice. 786-O is defective in VHL expression and used most commonly in RCC-focused research [21]. We confirmed the VHL expression using qRT-PCR (S1A Fig). In addition, as ccRCC exists in a clear-cell form, its morphological features are characterized by the accumulation of lipids and glycogen, indicating that gene mutations are related to major metabolic pathways, including lactate fermentation [22]. Accordingly, we have confirmed increased glucose uptake in ccRCC cells (Fig 2A), indicating that glycolysis is upregulated in ccRCC, as in other cancers, and that the enzymes catalyzing the change from glucose to pyruvate are naturally increased in RCC.

Most cancer cells produce ATP through lactate fermentation; the result of pyruvate conversion to lactate through the Warburg effect. In ccRCC, the majority of enzymes that feed metabolites from glycolysis, lipid metabolism, and from glutamine metabolism pathways into the tricarboxylic acid (TCA) cycle are decreased. Consistent with this down-regulation of the TCA cycle, OXPHOS activity is also decreased in ccRCC. A recent study reported that HIF-mediated inhibition of PGC1α suppressed mitochondrial respiration, and that after the expression of PGC1α was rescued, mitochondrial function was restored and tumor growth was suppressed [1]. The present experiments also revealed that PGC1α expression was restored after the overexpression of SIRT3 (Fig 1B and 1C), indicating that SIRT3 also affects mitochondrial function. Recent studies have found that enzymes involved in fatty acid oxidation are also decreased, while those involved in lipid storage are increased [6, 23]. Similarly, decreased tryptophan levels have also been reported, but downstream metabolites such as kynurenine, quinolinate, and the level of indoleamine 2,3-dioxygenase (IDO) (a tryptophan catalyst enzyme) were all increased in RCC [22].

Due to mitochondrial dysregulation, many metabolic changes occur in cancer. For RCC, these include: (1) a shift to anaerobic metabolism through HIF-dependent activation of many genes in the glycolytic pathway, (2) reductive carboxylation of glutamine-derived α-ketoglutarate as a carbon source instead of pyruvate in the TCA cycle, and (3) increased utilization of the pentose phosphate pathway [20]. In addition, levels of glucose transporter 1 (GLUT-1) are increased in ccRCC tumor tissue compared to normal control tissue, suggesting increased glucose uptake [24]. Moreover, metabolomic, proteomic, and transcriptomic studies show increased levels of glycolysis metabolites and enzymes in ccRCC cells, suggesting an upregulation of glucose utilization [6, 18, 25]. As shown here, SIRT3 OE reduced glucose uptake and L-lactate production (Fig 2A) in these cells. Both mitochondrial membrane potentials, complex I enzyme activity and their mass were enhanced (Fig 2B and 2C).On the other hand, the glycolysis antagonist fructose-1,6-bisphosphatase 1 (FBP1), and enzymes that catabolize pyruvate are depleted in ccRCC tumors, demonstrating that ccRCC relies on lactate fermentation [6, 26]. In mitochondria, fatty acyl-carnitine is converted back into fatty acyl-CoA, which undergoes fatty acid oxidation to form acetyl-coenzyme A (acetyl-CoA). Metabolomic studies of RCC show increased levels of long-chain fatty acids and decreased levels of enzymes involved in fatty acid oxidation using proteomics in ccRCC tissues [6, 18].

It is clear that mitochondria are directly involved in these cancer-cell features in addition to their providing more than 90% of the cellular energy as ’powerhouses of the cell’ [5]. Many factors (e.g., tissue type, tumor heterogeneity, tumor grade, and the microenvironment) regulate mitochondrial biogenesis in cancer. Indeed, mitochondria are also crucial mediators of apoptosis, the source of reactive oxygen species (ROS) generation, and energy production [17]. As shown here, SIRT3 can also restore mitochondrial functions, and improve the sensitivity of anti-cancer drugs in ccRCC cells (Fig 3). Recently, a role for SIRT3 in the regulation of mitochondrial biogenesis has emerged. SIRT3, localized in the mitochondrial matrix, controls many processes such as the TCA cycle, respiratory chain activity, and fatty acid oxidation [8, 27]. SIRT3 directly promotes mitochondrial metabolism through the modulation of multiple substrates [2, 28]. In the present study, expression of both PGC1α and PHD3 were increased after SIRT3 OE (Fig 1). We propose that SIRT3 is also used to maintain mitochondrial function and biogenesis.

Mitochondrial dysfunction is a main characteristic of ccRCC. SIRT3 is the major NAD+-dependent deacetylase in mitochondria and is downregulated in ccRCC tissues [29]. Many previous studies have established that SIRT3 regulates mitochondrial function and metabolism by increasing mitochondrial respiration, inhibiting glycolysis, and modulating cancer-cell proliferation [2, 28, 30]. Similarly, we confirmed decreased SIRT3 expression and decreased mitochondrial function in ccRCC cells. Previous studies have demonstrated decreased protein expression in mitochondria, indicating impaired mitochondrial biogenesis in ccRCC [4, 5, 7]. SIRT3 stimulates PGC1α gene expression through the activation of CREB phosphorylation [10]. Therefore, our observations that SIRT3 OE increased PGC1α expression (Fig 1B and 1C) and improved mitochondrial function (Fig 2) supports the idea that SIRT3 mediates the effects of PGC1α on mitochondrial metabolism. VHL expression of 786-O was not affected by SIRT3 overexpression, while increased in Caki-1 (S1A Fig). Thus, we suggest that SIRT3 improves mitochondrial function and biogenesis independent on VHL.

SIRT3 OE sensitizes cells to oxidative stress and promotes mitochondrial biogenesis by increasing both the expression and deacetylation of mitochondrial transcription factor A (TFAM) [2]. SIRT3 normalizes fatty acid oxidation by deacetylating long-chain acyl-CoA dehydrogenase (LCAD) and acetyl-CoA synthetase 2 (AceCS2); SIRT3 also accelerates glucose uptake by activating protein kinase B (Akt) [31]. In contrast, reduced SIRT3 expression decreases Complex I-V activities, oxygen consumption activity, mitochondrial membrane potential, and reduces both mitochondrial density and biogenesis [32].

Here, we found that SIRT3 OE in ccRCC cells resulted in the upregulation of mitochondrial genes related to their function. Not only was PGC1α (a transcriptional coactivator regulating mitochondrial biogenesis [33]) upregulated by SIRT3 OE, but also prolyl-4-hydroxylase domain 3 (PHD3), a factor required for PGC1α-induction [34]. In addition, SIRT3 OE led to a decrease in glucose uptake, an increase in mitochondrial membrane potential, and an increased quantity of healthy mitochondria. Confocal images also demonstrated increased numbers of mitochondria in SIRT3 OE ccRCC cells. PGC1α expression levels are often used as indicators of healthy mitochondria in tumors, with high PGC1α expression indicating a dependence on mitochondrial respiration [5, 14]. Our observations suggest that mitochondrial dysfunction may recover through the expression of SIRT3.

Both mitochondrial function and metabolic homeostasis are affected by RSV treatment in cells and tissue through RSV-induced expression of genes for oxidative phosphorylation and mitochondrial biogenesis. However, both PGC1α expression and PGC1α acetylation are unaltered by RSV treatment, despite the co-expression of PGC1α and SIRT1 [16]. We therefore tested whether RSV, coupled to increased SIRT3 expression, could modulate PGC1α function. Interestingly, PGC1α expression was significantly increased when SIRT3 OE cells were treated with RSV. Furthermore, we observed that SIRT3 OE ccRCC cells were more sensitive to anticancer drugs. Increased glycolysis and metabolic transformations are hallmarks of cancer cells, and the simple overexpression of SIRT3 improved cell sensitivity to our test drugs. Other studies have described the activation of key proteins such as SIRT1, SIRT3, and/or PGC1α following RSV treatment [16, 35]. Here, the combination of RSV treatment and SIRT3 OE induced both PGC1α activation and reduced glucose uptake compared to controls.

## Conclusion

The expression of SIRT3 improves mitochondrial function and biogenesis. Furthermore, the anti-proliferative effects of SIRT3 and RSV are increased in ccRCC through metabolic reprogramming. Thus, although further studies are required to establish its clinical potential, SIRT3 may represent an important factor for both cancer diagnosis and therapeutic development.

## Supporting information

S1 Fig VHL levels in ccRCC cells were measured. a. VHL mRNA levels were assessed using qRT-PCR after SIRT3 overexpression. Values are expressed as mean ± SEM (n = 4, per triplicate) and control-cell values were set to 1.
(PDF)

S2 Fig Normal tubule cells were not affected by SIRT3 overexpression. a. VHL mRNA levels were assessed using qRT-PCR after SIRT3 overexpression. Values are expressed as mean ± SEM (n = 3, per triplicate) and control-cell values were set to 1. b. Quantitative analysis of cellular glucose uptake (n = 6, per triplicate) and L-lactate production (n = 3, per triplicate). Values are expressed as mean ± SEM and control-cell values were set to 1. c. Mitochondrial membrane potentials were improved by SIRT3 overexpression. Data are expressed as mean ± SEM (n = 6, per triplicate) and control-cell values were set to 1. *p <0.05, **p <0.01.
(PDF)

S1 Raw images (PDF)

S1 Dataset (PDF)
