# Supplementary figures and images for: Mitochondrial metabolic reprogramming by SIRT3 regulation ameliorates drug resistance in renal cell carcinoma
Source: PLoS One. 2022 Jun 7;17(6):e0269432. doi: 10.1371/journal.pone.0269432 (PMC9173632; doi:10.1371/journal.pone.0269432)

a.

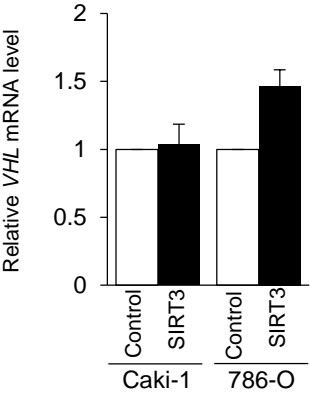

Supplement: S1 Fig — a. VHL mRNA levels were assessed using qRT-PCR after SIRT3 overexpression. Values are expressed as mean ± SEM (n = 4, per triplicate) and control-cell values were set to 1. (PDF) [file pone.0269432.s001.pdf]

a.

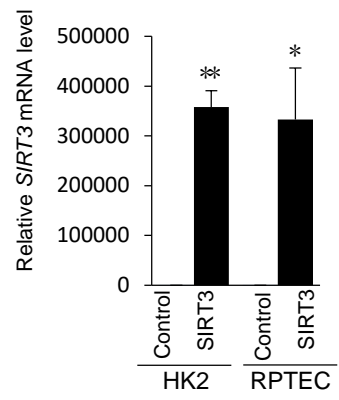

b.

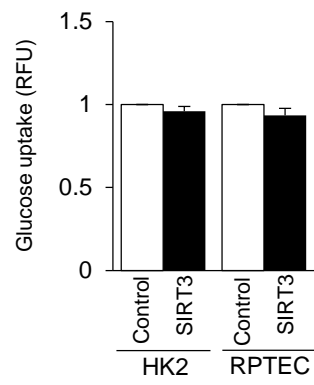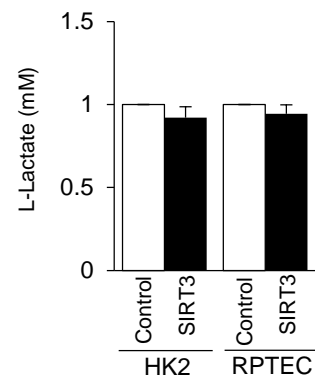

c.

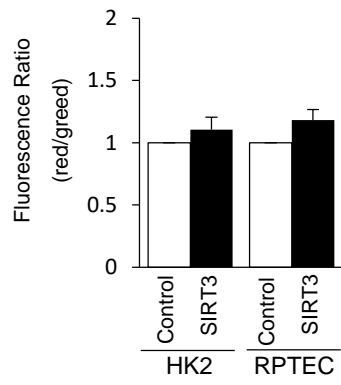

Supplement: S2 Fig — a. VHL mRNA levels were assessed using qRT-PCR after SIRT3 overexpression. Values are expressed as mean ± SEM (n = 3, per triplicate) and control-cell values were set to 1. b. Quantitative analysis of cellular glucose uptake (n = 6, per triplicate) and L-lactate production (n = 3, per triplicate). Values are expressed as mean ± SEM and control-cell values were set to 1. c. Mitochondrial membrane potentials were improved by SIRT3 overexpression. Data are expressed as mean ± SEM (n = 6, per triplicate) and control-cell values were set to 1. *p <0.05, **p <0.01. (PDF) [file pone.0269432.s002.pdf]

Figure 1

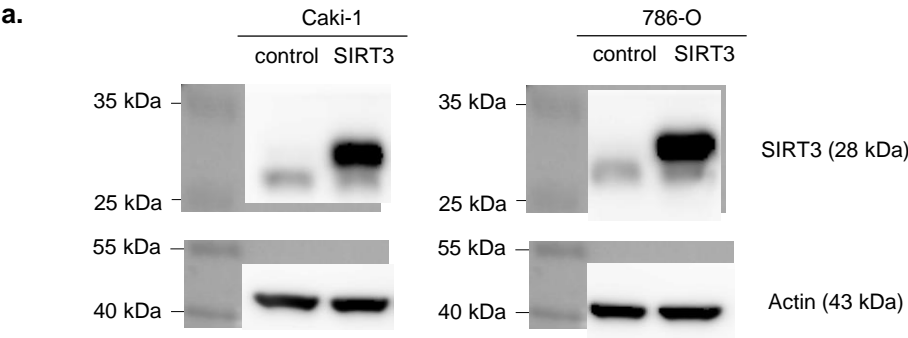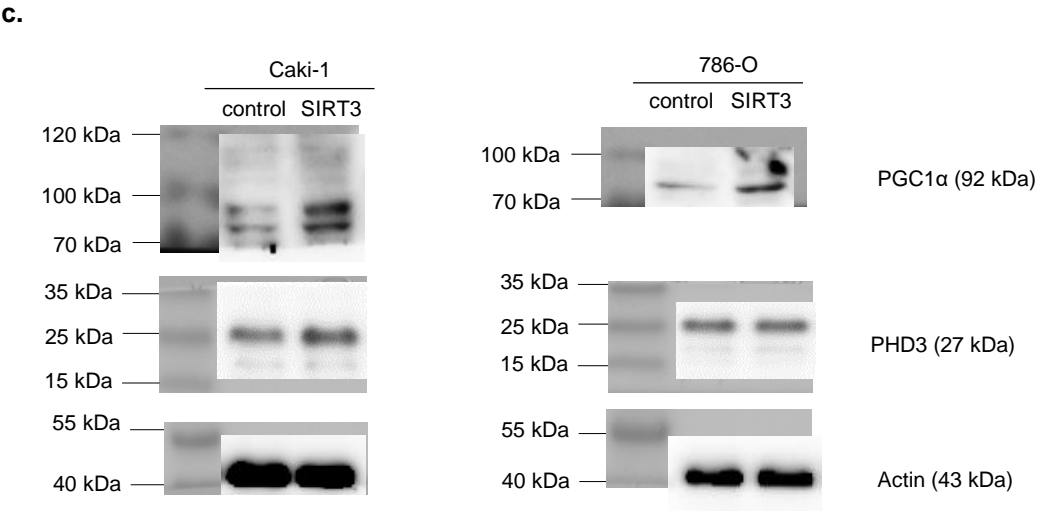

Supplement: S1 Raw images — (PDF) [file pone.0269432.s003.pdf]
