# Supplementary material for: Mitochondrial metabolic reprogramming by SIRT3 regulation ameliorates drug resistance in renal cell carcinoma
Source: PLoS One. 2022 Jun 7;17(6):e0269432. doi: 10.1371/journal.pone.0269432 (PMC9173632; doi:10.1371/journal.pone.0269432)
Supplement: S1 Dataset — (PDF) [file pone.0269432.s004.pdf]

Figure 1

a.

mRNA level

Caki-1, $p = 0.013$

	average	stdev	s.e
control	1	0	0
SIRT3	65521.809	13080.222	7551.8699

786-O, $p = 0.0064$

	average	stdev	s.e
control	1	0	0
SIRT3	10258.789	1434.0531	827.95092

protein

		average	stdev	s.e
786-O	actin	1	0	0
	sirt3	202.58742	55.779669	32.204407
caki-1	actin	1	0	0
	sirt3	124.14868	7.0974708	4.0977267

786-O, $p = 0.0037$

Caki-1, $p = 0.0004$

b.

Caki-1, $p = 0.033$

	average	stdev	s.e
control	1	0	0
PGC1a	3.3216186	0.7516589	0.4339705

$p = 0.022$

	average	stdev	s.e
control	1	0	0
PHD3	1.7251029	0.3446156	0.1989639

786-O, $p = 0.026$

	average	stdev	s.e
control	1	0	0
PGC1a	1.757356	0.3818409	0.220456

$p = 0.031$

	average	stdev	s.e
control	1	0	0
PHD3	1.3750954	0.1166304	0.0673366

c.

		average	stdev	s.e
Caki-1	control	1	0	0
	PGC1a	5.1583013	0.2745672	0.1585215
786-O	control	1	0	0
	PGC1a	2.8818332	0.0262735	0.015169

Caki-1, $p = 0.0002$

786-O, $p = 0.00000$

		average	stdev	s.e
Caki-1	control	1	0	0
	PHD3	2.7269209	0.5755098	0.3322707
786-O	control	1	0	0
	PHD3	1.3770003	0.1241452	0.0716752

Caki-1, $p = 0.0017$

786-O, $p = 0.011$

Figure 2

a.

Glucose uptake

Caki-1	pcDNA3-FL	SIRT3
average	1	0.6805774
stdev	0	0.1032737
s.e.	0	0.0421613

786-O	pcDNA3-FL	SIRT3
average	1	0.7661775
stdev	0	0.0523865
s.e.	0	0.0213867

Caki-1, $p = 0.0007$

786-O, $p = 0.0001$

Glycolysis (L-Lactate production)

Caki-1	pcDNA3-FL	SIRT3
average	1	0.8288667
stdev	0	0.054736
s.e.	0	0.0223459

786-O	pcDNA3-FL	SIRT3
average	1	0.8560228
stdev	0	0.047054
s.e.	0	0.0192097

Caki-1, $p = 0.0006$

786-O, $p = 0.0011$

b.

JC-1 assay

Caki-1	pcDNA3-FL	SIRT3
average	1	1.4567369
stdev	0	0.1614068
s.e.	0	0.0807034

786-O	pcDNA3-FL	SIRT3
average	1	1.6316807
stdev	0	0.2147221
s.e.	0	0.1073611

Caki-1, $p = 0.0013$

786-O, $p = 0.0095$

c.

OXPHOS (Complex I enzyme activity)

Caki-1	pcDNA3-FL	SIRT3
average	1	1.2379575
stdev	0	0.1903752
s.e.	0	0.0777203

786-O	pcDNA3-FL	SIRT3
average	1	1.3111573
stdev	0	0.0590135
s.e.	0	0.0240922

Caki-1, $p = 0.0280$

786-O, $p = 0.0000$

Figure 3

a.

mRNA level

Caki-1

**PGC1a**	average	stdev	s.e
Ctrl_DMSO	1	0	0
SIRT3_DMS	1	0	0
Ctrl_RSV	8.3206146	3.8431827	2.2188625
SIRT3_RSV	11.177307	1.8825924	1.0869152

control, p = 0.0019, SIRT3, p = 0.00000

786-O

**PGC1a**	average	stdev	s.e
Ctrl_DMSO	1	0	0
SIRT3_DMS	1	0	0
Ctrl_RSV	9.6328406	2.3344098	1.3477721
SIRT3_RSV	17.095767	5.3168401	3.069679

control, p = 0.00009, SIRT3, p = 0.00046

b.

Caki-1

Resveratrol, 50 uM p = 0.116, 100 uM p = 0.029, 150 uM p = 0.028

Caki-1	pcDNA3-FLAG				SIRT3			
	DMSO	50 uM	100 uM	150 uM	DMSO	50 uM	100 uM	150 uM
average	1	0.9421793	0.7435355	0.4571813	1	0.6983394	0.3793711	0.1915043
stdev	0	0.1347232	0.1147184	0.0363983	0	0.1606574	0.0169471	0.0260563
s.e.	0	0.0777825	0.0662327	0.0210146	0	0.0927556	0.0097844	0.0150436

Everolimus, 50 uM p = 0.0267, 100 uM p = 0.0278, 150 uM p = 0.0277

Caki-1	pcDNA3-FLAG				SIRT3			
	DMSO	50 uM	100 uM	150 uM	DMSO	50 uM	100 uM	150 uM
average	1	0.9299126	0.9148657	0.8026074	1	0.9142169	0.8572941	0.3022779
stdev	0	0.0056453	0.0615791	0.0509284	0	0.0649959	0.0250229	0.1242252
s.e.	0	0.0032593	0.0355527	0.0294035	0	0.0375254	0.014447	0.0717214

Temsirolimus, 50 uM p = 0.0277, 100 uM p = 0.0287, 150 uM p = 0.0278

Caki-1	pcDNA3-FLAG				SIRT3			
	DMSO	50 uM	100 uM	150 uM	DMSO	50 uM	100 uM	150 uM
average	1	0.9557494	0.818572	0.72912	1	0.7866882	0.6531305	0.4394688
stdev	0	0.0345401	0.0190772	0.0017077	0	0.0192826	0.0407857	0.0319741
s.e.	0	0.0199418	0.0110142	0.000986	0	0.0111328	0.0235476	0.0184603

786-O

Resveratrol, 50 uM p = 0.036, 100 uM p = 0.040, 150 uM p = 0.039

786-O	pcDNA3-FLAG				SIRT3			
	DMSO	50 uM	100 uM	150 uM	DMSO	50 uM	100 uM	150 uM
average	1	0.8769121	0.6850307	0.5423921	1	0.6753087	0.3767697	0.2132815
stdev	0	0.1316251	0.1200228	0.1719929	0	0.1707922	0.1310342	0.0368039
s.e.	0	0.0759938	0.0692952	0.0993002	0	0.0986069	0.0756526	0.0212487

Everolimus, 50 uM p = 0.097, 100 uM p = 0.097, 150 uM p = 0.74

786-O	pcDNA3-FLAG				SIRT3			
	DMSO	50 uM	100 uM	150 uM	DMSO	50 uM	100 uM	150 uM
average	1	0.9621502	0.8238689	0.5852656	1	0.8680749	0.5167826	0.2107237
stdev	0	0.0767814	0.1732152	0.0567945	0	0.1079668	0.1753424	0.1001022
s.e.	0	0.0443297	0.1000058	0.0327903	0	0.0623347	0.101234	0.057794

Temsirolimus, 50 uM p = 0.032, 100 uM p = 0.027, 150 uM p = 0.028

786-O	pcDNA3-FLAG				SIRT3			
	DMSO	50 uM	100 uM	150 uM	DMSO	50 uM	100 uM	150 uM
average	1	0.8976225	0.7235654	0.4888101	1	0.6611654	0.3818858	0.2124003
stdev	0	0.0650918	0.1061439	0.3351506	0	0.2657158	0.2535541	0.1333871
s.e.	0	0.0375807	0.0612822	0.1934993	0	0.1534111	0.1463895	0.0770111

c.

Glucose uptake

Caki-1	control			sirt3		
	RSV 150 uI	Ever. 10 uN	Tem. 20 uN	RSV 150 uI	Ever. 10 uN	Tem. 20 uN
average	1	1	1	0.8042124	0.7941723	0.7534379
stdev	0	0	0	0.024981	0.0326147	0.0557029
s.e	0	0	0	0.0124905	0.0163073	0.0278514

Caki-1,

RSV $p = 0.0005$

Ever. $p = 0.0011$

Tem. $p = 0.0030$

786-O	control			sirt3		
	RSV 150 uI	Ever. 10 uN	Tem. 20 uN	RSV 150 uI	Ever. 10 uN	Tem. 20 uN
average	1	1	1	0.8165781	0.872264	0.8387215
stdev	0	0	0	0.0223876	0.0386029	0.0860964
s.e	0	0	0	0.0111938	0.0193014	0.0430482

786-O,

RSV $p = 0.0005$

Ever. $p = 0.0070$

Tem. $p = 0.0332$

Glycolysis (L-Lactate production)

Caki-1	control			SIRT3		
	RSV 150 uI	Ever. 10 uN	Tem. 20 uN	RSV 150 uI	Ever. 10 uN	Tem. 20 uN
average	1	1	1	0.810346	0.8857246	0.7598341
stdev	0	0	0	0.050821	0.0279272	0.0344392
s.e	0	0	0	0.0293415	0.0161238	0.0198835

Caki-1,

RSV $p = 0.0231$

Ever. $p = 0.0021$

Tem. $p = 0.0068$

786-O	control			SIRT3		
	RSV 150 uI	Ever. 10 uN	Tem. 20 uN	RSV 150 uI	Ever. 10 uN	Tem. 20 uN
average	1	1	1	0.8055276	0.8857488	0.7535793
stdev	0	0	0	0.0388308	0.0427893	0.0594452
s.e	0	0	0	0.022419	0.0247044	0.0343207

786-O,

RSV $p = 0.0009$

Ever. $p = 0.0099$

Tem. $p = 0.0189$

d.

Caki-1	control			SIRT3		
	RSV 150 uI	Ever. 10 uN	Tem. 20 uN	RSV 150 uI	Ever. 10 uN	Tem. 20 uN
average	1	1	1	1.4453138	1.3953667	1.3953156
stdev	0	0	0	0.0591641	0.0821746	0.0897674
s.e	0	0	0	0.0341584	0.0474435	0.0518273

Caki-1,

RSV $p = 0.0002$

Ever. $p = 0.0141$

Tem. $p = 0.0168$

786-O	control			SIRT3		
	RSV 150 uI	Ever. 10 uN	Tem. 20 uN	RSV 150 uI	Ever. 10 uN	Tem. 20 uN
average	1	1	1	1.3605179	1.3221624	1.360974
stdev	0	0	0	0.0898945	0.0531396	0.0897078
s.e	0	0	0	0.0519006	0.0306802	0.0517928

786-O,

RSV $p = 0.0022$

Ever. $p = 0.0089$

Tem. $p = 0.0200$

Supplementary Figure 1

a.

mRNA level

Caki-1, $p = 0.0897$

	average	stdev	s.e
control	1	0	0
SIRT3	1.4656507	0.2594148	0.1497732

786-O, $p = 0.8099$

	average	stdev	s.e
control	1	0	0
SIRT3	1.0343773	0.2081226	0.1201597

Supplementary Figure 2

a.

HK2	pcDNA3-FL	SIRT3
**average**	1	357933.11
**stdev**	0	32752.036
**s.e.**	0	32752.036

HK2, $p = 0.0073$

RPTEC	pcDNA3-FL	SIRT3
**average**	1	333148.45
**stdev**	0	103418.45
**s.e.**	0	103418.45

RPTEC, $p = 0.0127$

b.

Glucose uptake

HK2	pcDNA3-FL	SIRT3
**average**	1	0.9585903
**stdev**	0	0.0714777
**s.e.**	0	0.0291806

HK2, $p = 0.3305$

RPTEC	pcDNA3-FL	SIRT3
**average**	1	0.934798
**stdev**	0	0.1013563
**s.e.**	0	0.0413785

RPTEC, $p = 0.0790$

Glycolysis (L-Lactate production)

HK2	pcDNA3-FL	SIRT3
**average**	1	0.9218266
**stdev**	0	0.1594685
**s.e.**	0	0.0651027

HK2, $p = 0.8635$

RPTEC	pcDNA3-FL	SIRT3
**average**	1	0.9442532
**stdev**	0	0.1298666
**s.e.**	0	0.0530178

RPTEC, $p = 0.8044$

c.

HK2	pcDNA3-FL	SIRT3
**average**	1	1.1049046
**stdev**	0	0.1981714
**s.e.**	0	0.0990857

HK2, $p = 0.3674$

RPTEC	pcDNA3-FL	SIRT3
**average**	1	1.1815927
**stdev**	0	0.1718177
**s.e.**	0	0.0859089

RPTEC, $p = 0.1249$
